# Targeting HIV-1 RNase H: *N’*-(2-Hydroxy-benzylidene)-3,4,5-Trihydroxybenzoylhydrazone as Selective Inhibitor Active against NNRTIs-Resistant Variants

**DOI:** 10.3390/v12070729

**Published:** 2020-07-06

**Authors:** Angela Corona, Ester Ballana, Simona Distinto, Dominga Rogolino, Claudia Del Vecchio, Mauro Carcelli, Roger Badia, Eva Riveira-Muñoz, Francesca Esposito, Cristina Parolin, José A. Esté, Nicole Grandi, Enzo Tramontano

**Affiliations:** 1 Department of Life and Environmental Sciences University of Cagliari, Cittadella Universitaria di Monserrato, 09042 Monserrato, Italy; s.distinto@unica.it (S.D.); francescaesposito@unica.it (F.E.); nicole.grandi@unica.it (N.G.); tramon@unica.it (E.T.); 2 AIDS Research Institute—IrsiCaixa, 08916 Badalona, Spain; eballana@irsicaixa.es (E.B.); rbadia@irsicaixa.es (R.B.); eriveira@irsicaixa.es (E.R.-M.); jaeste@cienciatraducida.com (J.A.E.); 3 Department of Chemistry, Life Sciences and Environmental Sustainability, University of Parma, 43124 Parma, Italy; dominga.rogolino@unipr.it (D.R.); mauro.carcelli@unipr.it (M.C.); 4 Department of Molecular Medicine, University of Padova, 35122 Padova, Italy; claudia.delvecchio@unipd.it (C.D.V.); cristina.parolin@unipd.it (C.P.); 5 CienciaTraducida, 08391 Barcelona, Spain; 6 Istituto di Ricerca Genetica e Biomedica, Consiglio Nazionale delle Ricerche (CNR), 09042 Monserrato, Cagliari, Italy

**Keywords:** HIV-1, antivirals, ribonuclease H, RNase H, *N*-acylhydrazone, RT

## Abstract

HIV-1 infection requires life-long treatment and with 2.1 million new infections/year, faces the challenge of an increased rate of transmitted drug-resistant mutations. Therefore, a constant and timely effort is needed to identify new HIV-1 inhibitors active against drug-resistant variants. The ribonuclease H (RNase H) activity of HIV-1 reverse transcriptase (RT) is a very promising target, but to date, still lacks an efficient inhibitor. Here, we characterize the mode of action of *N’*-(2-hydroxy-benzylidene)-3,4,5-trihydroxybenzoylhydrazone (compound **13**), an *N*-acylhydrazone derivative that inhibited viral replication (EC_50_ = 10 µM), while retaining full potency against the NNRTI-resistant double mutant K103N-Y181C virus. Time-of-addition and biochemical assays showed that compound **13** targeted the reverse-transcription step in cell-based assays and inhibited the RT-associated RNase H function, being >20-fold less potent against the RT polymerase activity. Docking calculations revealed that compound **13** binds within the RNase H domain in a position different from other selective RNase H inhibitors; site-directed mutagenesis studies revealed interactions with conserved amino acid within the RNase H domain, suggesting that compound **13** can be taken as starting point to generate a new series of more potent RNase H selective inhibitors active against circulating drug-resistant variants.

## 1. Introduction

The number of people worldwide infected with HIV-1 in 2019 has been estimated to be around 37.9 million [1]. Optimum treatment regimens consist of a combination of two [2] or more antiretrovirals with a different mode of action, that allows the suppression of viremia to undetectable levels, giving a life-expectation comparable to uninfected people [3,4]. However, despite promising efforts, presently approved treatments do not allow immunization of people [5] or the eradication of the infection [6], requiring life-long treatment with optimal adherence. At the moment, only 64.6% of HIV-1 infected people are accessing antiretroviral therapy [1]. Lack of treatment adherence and suboptimal coverage of the infected population cause the emergence of drug-resistant variants (DRV) and treatment failure [7]. DRV transmission narrows the options for treatment and is a major concern that requires an optimized therapy [8,9,10]. Thus, there is a constant need to explore new drugs and new targets for salvage therapy.

Among the viral encoded enzymatic functions, the HIV-1 RT-associated ribonuclease H (RNase H) represents the sole target for which an effective antiviral drug has not yet been approved. HIV-1 RNase H function is essential for viral replication [11] and is characterized by a highly conserved catalytic domain, with Asp–Asp–Glu (DDE) motif, essential for coordination of two Mg^2+^ or Mn^2+^ ions and by a tertiary structure [12] conserved among *Ortervirales* [13,14,15,16,17].

Two decades of research has allowed the identification of many compounds able to block HIV-1 RNase H activity. These compounds may be grouped in two main families according to their binding mode [18,19,20,21]: (i) active site inhibitors, which chelate the two Mg^2+^ within the active site; and (ii) RNase H allosteric inhibitors. The most potent inhibitors belong to the first class [22]. Active-site inhibitors take advantage of the driving force represented by their chelating moiety to stably interact with the catalytic core domain [22,23]. Despite notable anti-HIV potency of active-site inhibitors, off-target toxicity specific to this mode of action [24] and an unclear competition with the substrate [25] have precluded their further development as therapeutic agents.

Attempts to target highly conserved residues in the RNase H domain have been successfully shown [26,27,28]. Given the structural similarities and overlaps with the HIV-1 IN catalytic core, many of the identified RNase H active-site inhibitors exhibit dual anti-RNase H and anti-integrase (IN) activity [20,29]. Nevertheless, compounds with partial or even total selectivity for HIV-1 RNase H function has been obtained while retained full potency of inhibition against multidrug-resistant RTs [10].

**Figure 1:**
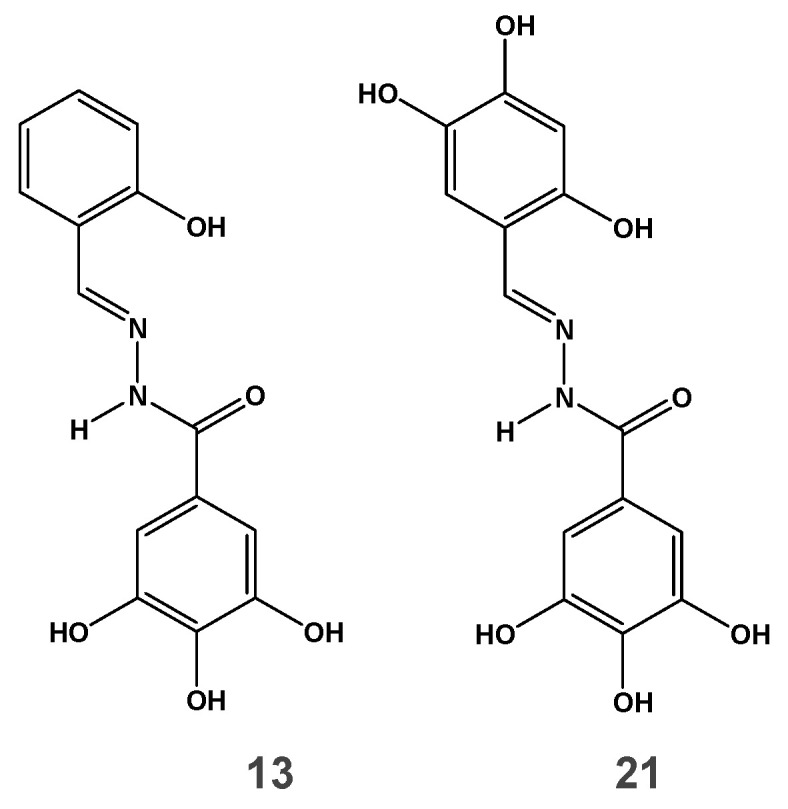
Structure of *N*-acylhydrazone analogs.

We previously investigated a series of *N*-acylhydrazone analogs to identify dual inhibitors of both HIV IN and RNase H [30]. Among them several compounds inhibited the RT-associated RNase H function in the low micromolar range, being inactive against HIV-1 IN. In the present work, we aim to dissect the mode of action of these inhibitors, laying the bases for further development as lead compounds. To this aim, two among previously reported compounds were chosen: compound **13** (Figure 1) as an example of active-site inhibitor selective for RNase H in enzymatic assays [30] and compound **21** (Figure 1), that inhibited also HIV-1 IN catalytic activity [30] as counterpart example of an RNase H/IN dual inhibitor.

Time-of-addition and antiviral assays showed that compound **13** blocks HIV-1 replication in cell culture by targeting the RNase H function during the reverse-transcription step and, importantly, retained full potency of inhibition against a non-nucleoside RT inhibitor (NNRTI) drug-resistant strain. The combined results of computational modeling and site-directed mutagenesis revealed that compound **13** interacts with the RNase H active site, coordinating the ionic cofactors, binding in an orientation different from the known inhibitor **RDS1759 [26]**, and establishing additional interactions with residues of the RNase H domain. The analysis of the RNase H domain nucleotide sequences revealed the presence of highly conserved amino acid residues, also part of the RNase H primer grip motif interacting with compound **13** [31]. This demonstrated that amino acid residues involved in compound **13** interaction with RT are highly conserved and give insights for the optimization of the lead.

## 2. Materials and0 Methods

### 2.1. Biology

#### 2.1.1. Cells Reagents and Viruses

Compounds **13** and **21** were synthesized as previously reported [30]. The inhibitors: azidothymidine (AZT); efavirenz (EFV); nevirapine (NVP) and raltegravir (RAL) were purchased form Sigma-Aldrich (St. Louis, MO, USA). RNase H inhibitor was kindly provided by Prof. Roberto Di Santo. MT4 (ECACC 08081402) and HEK 293 T cells (ATCC® CRL-3216TM) were cultured in RPMI 1640 medium and Dulbecco’s modified Eagle’s medium (DMEM) (Gibco, Waltham, MA, USA), respectively. Both media were supplemented with 10% fetal calf serum (FCS) (Gibco, Waltham, MA, USA) and 1% penicillin/streptomycin. Stocks of HIV-1 NL4-3 strain were prepared by transfecting 293 T cells with the HIV-1 pNL4-3. The VSV-pseudotyped, NL4-3–GFP expressing virus (VSV/HIV-1_GFP_) was generated as reported [32].

#### 2.1.2. Antiviral and Cytotoxicity Assay

The antiviral assay was performed as previously described [33,34]. Briefly, drug-mediated inhibition of virus-induced cytotoxicity was assayed in MT4cells at 120 h post infection. Triplicate wells of 96-well plates containing serial dilutions of drugs were seeded with 3 × 10^4^ MT4 cells and infected with HIV-1 NL4.3 wt or with the HIV-1 NL4-3 K103N-Y181C strain at multiplicity of infection (MOI) of 0.003 or 100 culture infective dose (CCID_50_) calculated using the Reed and Muench method [35]. Five days post infection, 20 μL 3-(4,5-dimethylthiazol-2-yl)-2,5-diphenyltetrazolium bromide (MTT) per well were added. After 1 h of incubation at 37 °C in a humidified atmosphere with 5% CO_2_, 150 μL of supernatant were carefully removed and 100 μL of acidified Triton X-100 isopropanol solution were added to each well and allow the complete dissolution of the formazan crystals by placing the plates on a vibrating platform shaker for 10 min.

The amount of formazan produced was quantified spectrophotometrically at 550/620 nm. The 50% effective concentration (EC_50_) and 50% cytotoxic concentration (CC_50_) values were calculated for each compound by applying a nonlinear regression with log dose vs. normalized response to the data.

#### 2.1.3. Time of Addition Assay

Time-of-addition assay was performed according to [36] using the VSV/HIV-1_GFP_ virus. The MT4 cells/well were pelleted and resuspended with RPMI containing VSV/HIV-1_GFP_ at a MOI of 0.2, centrifuged for 1 h at 1200× *g* at 15 °C and washed 2 times with cold PBS. A total of 1 × 10^4^ infected cells were seeded (100 μL) in a 96-well plate round bottom, after addition of 50 μL of RPMI to the whole plate and 50 μL 4X-dilutions of drugs to the first column, representing time 0. Drugs were added at 11 time-points post infection (1 h, 1.5 h, 2 h, 2.5 h, 3 h, 4 h, 5 h, 6 h, 7 h, 8 h, 24 h). Drugs were used at the following final concentrations: AZT—3.7 μM; NVP—3.7 μM; EFV—0.3 μM; RAL—2.25 μM; compound **13**—25 μM; compound **21**—25 μM; compound **21**—25 μM. Twenty-four hours post infection, cells were washed twice with PBS and fixed with 1% formaldehyde. GPF expression was quantified by fluorescence-activated cell sorting (FACS) with a LSRII flow cytometer (BD Biosciences, San José, CA, USA), recording FSC, SSC and FITC data. Data were analyzed with FlowJo software version10.6.1 (Tree Star Inc., Ashland, OR, USA).

### 2.2. Molecular Modeling

#### 2.2.1. Ligand Preparation

The ligand was built within the Mestro platform, the geometry was optimized with MacroModel (Schrödinger, LLC, New York, NY, USA, 2020) [37] using the Merck molecular force fields (MMFFs) [38], the GB/SA solvation model [39] and the Polak–Ribière conjugate gradient (PRCG) method and a convergence threshold of 0.05 kJ/(molÅ).

#### 2.2.2. Protein Preparation

Starting crystal coordinates of the complex RT–RNase H inhibitor was downloaded from the Protein Data Bank (http://www.rcsb.org/) pdb accession code 3LP2 [40]. Afterward, the protein was prepared using the Schrödinger protein preparation wizard [41]: hydrogen atoms were added to the system. Partial atomic charges were assigned according to the optimized potential for liquid simulations (OPLS-2005) force field [42]. A restrained minimization was performed to optimize hydrogen atoms and to remove eventually any high-energy contacts or distorted bonds, angles and dihedrals with to converge atoms to RMSD 0.3.

#### 2.2.3. Docking and Post-Docking

Compound **13** was docked into wt RTs through QM-polarized ligand docking protocol applying default settings [43]. In order to better take into account the induced fit phenomena, the most energy favored generated complexes were fully optimized with the OPLS force field in GB/SA implicit water [39], setting a 10,000-step-interaction analysis with Polak–Ribière conjugate gradient (PRCG) method and a convergence criterion of 0.1 kJ/(molÅ). The resulting complexes were considered for the binding modes graphical analysis with Pymol (Schrödinger, LLC, New York, NY, USA, 2020) [44] and Mestro (Schrödinger, LLC, New York, NY, USA, 2020) [41].

### 2.3. Pharmacophore Model Generation

All ligands reported in previous work [30] were modelled by means of LigandScout software (ver. 4.4) (Inte: Ligand, Vienna, Austria) [45,46]. Conformational analysis was performed before the alignment process applying best setting (i.e., max 200 conformations). Then compounds were aligned and the ligand-based share pharmacophore was automatically generated considering both scoring functions: pharmacophore fit and atom overlap.

### 2.4. Molecular Biology

#### 2.4.1. Expression and Purification of Recombinant HIV-1 RTs Group M Subtype B wt and Mutants

The p6HRT-prot vector was kindly provided by Dr. Stuart Le Grice Laboratory. Heterodimeric RT was expressed and purified essentially as described [47]. Briefly, *Escherichia coli* strain M15 containing the p6HRT-prot vector were grown up to an OD600 of 0.7 and induced with isopropyl β-D-1-thiogalactopyranoside (IPTG) (Sigma-Aldrich, St. Louis, MO, USA) 1.7 mM for 4 h at 37 °C. Cells were pelleted and lysed with 50-mM sodium phosphate pH 7.8, 0.5-mg/mL lysozyme, sonicated and centrifuged at 30,000× *g* for 1 h. The proteins were expressed and purified by a combination of affinity and ion-exchange chromatography. First the supernatant was loaded into a Ni^2+^ Sepharose (GE Healthcare, Chicago, IL, USA) with loading buffer (50-mM sodium phosphate pH 7.8, 0.3-M NaCl, 10% glycerol, 10-mM imidazole) and washed thoroughly with the same buffer at 80-mM imidazole. RTs were eluted with a gradient of up to 0.5-M imidazole. Fractions were collected; protein purity was checked by SDS-PAGE and found to be higher than 90%. RT containing fractions were pooled and diluted 1:1 with dilute buffer (50-mM sodium phosphate pH 7.0, 10% glycerol) then loaded into a Hi-trap Heparin HP (GE Healthcare, Chicago, IL, USA) preequilibrated with 10-column volume of loading buffer 2 (50-mM sodium phosphate pH 7.0, 10% glycerol, 150-mM NaCl). The column was then washed with loading buffer 2 and RT was gradient-eluted with elute buffer 2 (50-mM sodium phosphate pH 7.0, 10% glycerol, 150-mM NaCl). Fractions were collected, the protein was dialyzed and stored in buffer containing 50-mM Tris HCl pH 7.0, 25-mM NaCl, 1-mM EDTA, 50% glycerol. Catalytic activities and protein concentration were determined. Enzyme-containing fractions were pooled, and aliquots were stored at −80 °C.

#### 2.4.2. Site-Directed Mutagenesis

Amino acid substitutions were introduced into the p66 HIV-1 RT subunit coded in a p6HRT-prot plasmid using the QuikChange protocol (Agilent Technologies, Inc., Santa Clara, CA, USA).

#### 2.4.3. HIV-1 DNA Polymerase-Independent RNase H Activity Determination

The RT-associated RNase H activity of wt and mutated HIV RTs was measured as described [48], in 100 µL reaction volume containing 50 mM Tris HCl pH 7.8, 6-mM MgCl_2_, 1-mM dithiothreitol (DTT), 80-mM KCl, hybrid RNA/DNA (5′-GTTTTCTTTTCCCCCCTGAC-3′-fluorescein, 5′-CAAAAGAAAAGGGGGGACUG-3′-dabcyl, from Metabion, Planegg, Germany) and different amounts of enzymes according to a linear range of dose–response curve (60 ng of wt RT; 60 ng R448A RT; 160 ng R557A RT; 600 ng Q475A RT). The reaction mixture was incubated for 1 h at 37 °C, the reaction was stopped by addition of EDTA and products were measured with a Victor 3 (PerkinElmer, Waltham, MA, USA) at 490/528 nm. Mean ± standard deviation of 50% inhibitory concentration (IC_50_) values was determined and p-values were calculated between IC_50_ value against the wt and IC_50_ value against the mutants by paired, two-tailed *t*-tests using GraphPad Prism 6.01 software (GraphPad Software, Inc.; San Diego, CA, USA). Figures were made with GraphPad Prism 6 version 6.01.

#### 2.4.4. HIV-1 RNA-Dependent DNA Polymerase (RDDP) Activity Determination

The HIV-1 RT-associated RDDP activity was measured as described [49], in 25 µL volume containing 60-mM Tris-HCl pH 8.1, 8-mM MgCl_2_, 60-mM KCl, 13-mM DTT, poly(A)-oligo(dT), 100-µM dTTP and 6 ng wt RT. After enzyme addition, the reaction mixture was incubated for 30 min at 37 °C and enzymatic reaction was stopped by addition of EDTA. Reaction products were detected by PicoGreen addition and measured with a Victor 3 (PerkinElmer, Waltham, MA, USA) at 502/523 nm. Figures were made with GraphPad Prism 6 version 6.01.

### 2.5. Genetic Analysis

A nucleotide sequence collection including a total of 15.865 RT isolates was downloaded from the HIV-1 Single Genome Sequence (SGS) Database of the Stanford University HIV database [50,51] (https://hivdb.stanford.edu/project/sgs/). RT nucleotide sequences were aligned using Geneious software [52] implemented with MAFFT aligner [53], and RH nucleotide portion was extracted and translated to obtain the deriving RH proteins. The RT isolates devoid of the RNase H portions were excluded, as well as those including only a partial RNase H domain or showing internal frameshifts and stop codons, ending up with a total of 5798 aligned RH proteins. The latter were analyzed with respect to HIV-1 strain NL4.3 RT–RNase H, used as reference protein, indicating for each amino acid residue the percentage of conservation. A logo representing the degree of conservation of each amino acidic position was also produced from the RNase H domain protein alignment with the tool WebLogo 3 (http://weblogo.threeplusone.com/create.cgi).

## 3. Results

To determine the mode of action of *N*-acylhydrazone analogs that displayed selective inhibition of HIV-1 RT RNase H associated function and to acquire information useful to optimize the scaffold, a series of cell -based and enzymatic assays were performed on compound **13**.

### 3.1. Antiviral Activity of N-acylhydrazone Analogs against wt and NNRTI-Resistant HIV-1 Strains

Antiviral activity and cytotoxicity of compounds **13** and **21** were determined at 5 days on MT4 cells against HIV-1 NL4.3 wt strain and HIV-1 NL4.3 carrying the K103N-Y181C double mutation, commonly selected in patients and that confers resistance to non-nucleoside RT inhibitors (NNRTIs). The nucleoside inhibitor AZT, the NNRTIs EFV and NVP, the IN inhibitor RAL and the RNase H inhibitor **RDS1759** were used as positive controls (Table 1). Both *N*-acylhydrazone derivatives inhibited viral replication retaining similar potency of inhibition against the NNRTI-resistant double mutant NL4-3 K103N-Y181C virus, similarly to AZT and RAL and the RNase H inhibitor **RDS1759**, while the NNRTIs lost their antiviral activity. Unfortunately, the cytotoxicity showed by the compounds determined a selectivity index below 10.

### 3.2. Analysis of Compound ***13*** Target of Inhibition by Time-of-Addition and Comparative Inhibition of HIV-1 RT Associated Activities

**Figure 2:**
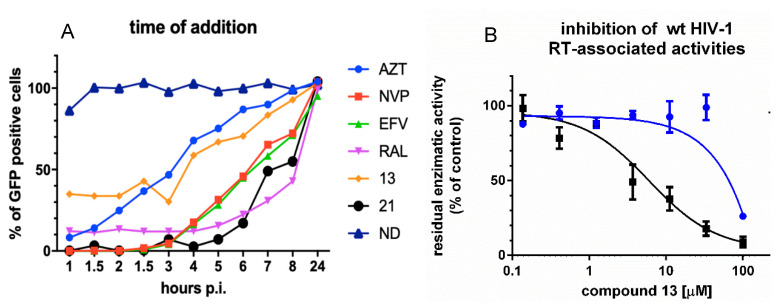
Analysis of compound **13** target of inhibition. (**A**) Time-of-addition. Drugs were used at the following final concentrations: AZT 3.7 μM; NVP 3.7 μM; EFV 0.3 μM; RAL 2 25 μM; compound **13** 25 μM; compound **21** 25 μM. Twenty-four hours post infection GFP expression was quantified by FACS. A representative assay of two is shown; (**B**) comparative inhibition of HIV-1 RT-associated activities. RT-associated RNase H activity black line, RDDP blue line. Assays were run in duplicate; the graph represents values the mean and standard deviation of two independent experiments.

To confirm that compound **13** was selectively targeting the RT-associated RNase H function during HIV-1 replication, we performed a time-of-addition of compound **13** at 25 μM using the RNase H/IN dual inhibitor compound **21**, the nucleoside inhibitor AZT, the NNRTIs EFV and NVP and the IN inhibitor RAL as internal controls (Figure 2A). The two *N*-acylhydrazones showed a very different behavior: compound **13** lost activity 6 h after infection, in a time–window between AZT and EFV/NVP, in agreement with the hypothesis of targeting the retrotranscription step of the replicative viral cycle consistently with the inactivity against HIV-1 IN previously reported [30] and suggesting a mode of action different from nucleoside analogs and NNRTIs. Differently, compound **21** retained its potency over time, together with the IN inhibitor RAL affecting also the integration step, accordingly with the previously reported inhibition of IN catalytic activity [30].

Once shown that compound **13** is indeed selectively inhibiting the viral genome reverse transcription, since several RNase H inhibitors were demonstrated to be able to inhibit both RT-associated functions [20,54], we asked whether compound **13** could affect also the RT-associated RNA-dependent DNA polymerase (RDDP) activity. Hence, a comparative inhibition of both HIV-1 RT associated activities was performed (Figure 2B). To overcome any possibility of overestimating the potency of inhibition, the experiments were performed in conditions of competition between the compounds and the substrate, since both inhibitor and substrate were added in solution before the enzyme. Results showed that compound **13** inhibited RT-associated RNase H activity with an IC_50_ value of 3.6 μM, while only a residual inhibition of the RDDP activity was observed (IC_50_ value of 81 μM), with no inhibitory effect at 25 μM, clearly indicating that the inhibition observed at 25 μM in the cell-based time-of-addition experiment and the inhibition of viral replication at 5 days (EC_50_ 10.1 µM) was specifically due to its inhibition of the HIV-1 RT-associated RNase H function.

### 3.3. Analysis of the Binding of Compound ***13*** within the HIV-1 RT RNase H Domain

In order to have insights on the binding mode of compound **13** within the HIV-1 RT RNase H domain, docking studies were performed.

**Figure 3:**
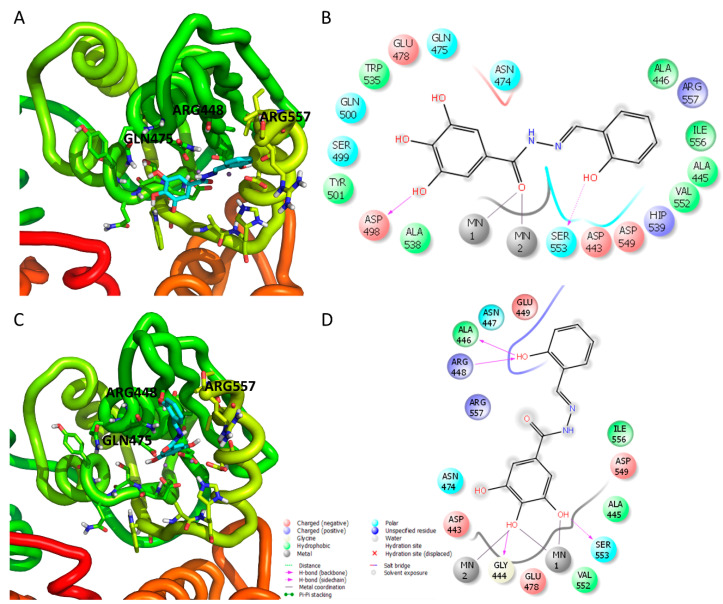
Putative binding mode of compound **13** within the HIV-1 RT RNase H domain. (**A**–**C**) Compound **13** in cyan depicted in the wt-RT: p66 in green, p51 in orange; (**B**–**D**) 2D representation of compound **13** in complex with wt RT with indication of binding pocket interacting residues.

**Figure 4:**
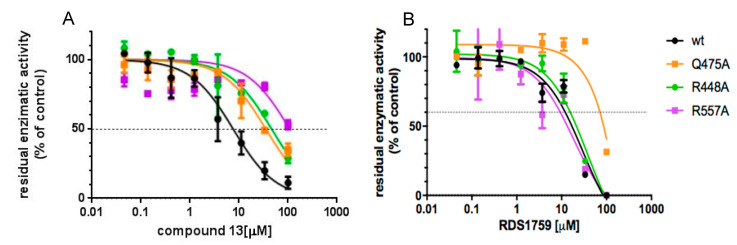
Inhibitory profile of compound **13** against the HIV-1 RT RNase H function. (**A**) Compound **13** inhibition of HIV-1 RT RNase H activities; (**B**) **RDS1759** inhibition of HIV-1 RT RNase H activities. Data represent the mean and SD of two experiments.

Docking calculations showed that compound **13** binds within the RNase H domain in two possible orientations (Figure 3). In the first one (Figure 3A,B), compound **13** coordinates the two Mn^2+^ ions with the carbonyl moiety of the acylhydrazone group, establishing additional interactions with residues Ser553, Asp498, Asn474, Gln475, Glu478. In the second binding mode (Figure 3C,D) compound **13** coordinates the two Mn^2+^ cofactors with the pyrogallol moiety serving as a chelating motif, while the remaining atoms of the molecule accommodate between alpha-helix E (in yellow) and beta-sheet 1 (in green), interacting with charged and polar residues Asp443, Glu449, Glu478, Arg557, Asn474, Arg557, establishing further hydrogen bonds with the side chain of Gly444, Ala446 and Arg448 (Figure 4B). This last orientation of the chelating portion is different from the one reported for the selective RNase H inhibitor **RDS1759,** but similar to the chelating portion of other compounds as beta-tuhjaplicinol (pdb 3I1G) [55] and 2-hydroxyisoquinoline-1,3-dione (pdb code 5UV5) [56], while the binding mode exhibited by the tail portion of compound **13** significantly differs from all of them.

### 3.4. Site-Directed Mutagenesis Studies Detailing the Binding Mode of Compound ***13***

To confirm the model suggested by the docking calculation—and to determine which of the two binding orientations was the most probable—site-directed mutagenesis studies were performed on amino acid residues involved in critical interactions with either compound **13** or **RDS1759** hypothesized to bind to RT differently from compound **13**. Among the suggested residues, Arg448, Gln475 and Arg557 were chosen as the most significant to assess the binding mode. In particular, Arg448 plays a discriminant role since it is involved in the interaction between compound **13** and the RNase H domain in the sole second orientation, while both Arg448 and Arg557 would discriminate the differences with the known inhibitor **RDS1759** that does not interact with that portion of the HIV-1 RT RNase H domain [26]. Hence, the single point mutations R448A, Q475A and R557A were independently introduced in the HIV-1 *pol* and mutant R448A-, Q475A- and R557A-RT were expressed and purified. Compound **13** and **RDS1759** inhibitory profile of the HIV-1 RT RNase H function of the enzymes were determined in parallel with the wt (Figure 4). Results showed that compound **13** inhibited the wt enzyme with an IC_50_ value of 6.9 μM, with a consistent decrease in potency against all the mutated enzymes (Figure 4A). The IC_50_ value increases of 5.2-fold against Q475A (*p*-value = 0.0003); 9.2-fold against R448A (*p*-value <0.0001) and more than 16.2-fold against R557A (*p*-value < 0.0001). Differently, RDS1759 potency of inhibition was not significantly affected against RTs carrying the R448A or R557A mutations as compared to the wt RT (p-values equal to 0.5545 and 0.9682, respectively) while it significantly lost its potency when tested on the Q475A RT (*p*-value = 0.0008). Overall, the data confirm the binding pose number 2 suggested by docking calculation.

### 3.5. Genetic Analysis

**Figure 5:**
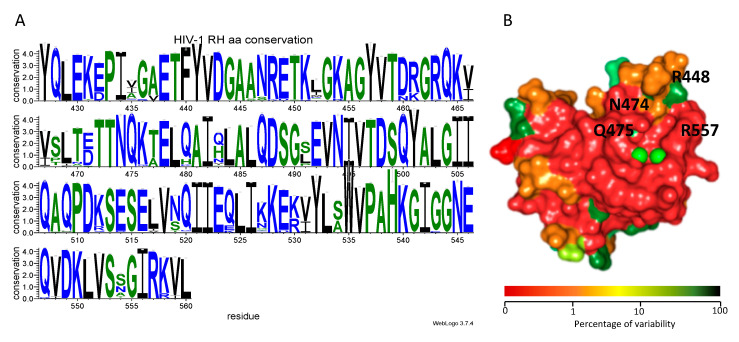
Conservation profile of HIV-1 RT RNase H domain. (**A**) Logo of degree of conservation of amino acid residues: blue hydrophobic lateral chains, green neutral lateral chains, black hydrophilic lateral chains; (**B**) color scheme of the percentage of variability of HIV-1 RT RNase H domain (PDB code 3K2P). The color scale of the percentage of variability goes form red (low percentage of variation) to green (high percentage of variation).

To establish whether the residues involved in the interaction between the HIV-1 RNase H domain and compound **13** were conserved, we evaluated the existing variability of HIV-1 RT RNase H domain among circulating HIV-1 variants, using a nucleotide collection including a total of 15.865 RT isolates from the HIV-1 Single Genome Sequence (SGS) Database of the Stanford University HIV database [51]. After alignment of the sequences, we retained for analysis only the ones containing a complete HIV-1 RT RNase H domain, for a total number of 5798 isolates. The latter were analyzed using as reference protein HIV-1 strain NL4.3 RNase H, indicating for each amino acid residue the percentage of conservation (Table S1, Supplementary Materials). A logo representing the degree of conservation of each amino acid position was produced (Figure 5A). Results showed that the HIV-1 RT RNase H domain has a very conserved structure (Figure 5A, PDB code 3K2P), with a number of patterns of residues that display a variability lower than 0.5% (amino acid residues: 427–430; 438–445; 472–475; 485–490; 497–501; 535–542; 549–557) with Arg557 and Gln475 showing a 0% variability and Asn474 and Gln444 showing a 0.1% variability. Interestingly, residues Arg448 and Glu449 also displayed a good degree of conservation (96.70% and 95.40%, respectively), with Arg449 being replaced by Lys in the 3.3% of the cases and Glu449 being replaced by Asp in 4.6% of the cases, suggesting strong preservation of their structural functions and of the spatial characteristic needed for binding of compound **13**.

### 3.6. Pharmacophore Model

Considering the biologic data previously reported for compounds **1**–**23** (Table S2) [30] and aiming at rationalizing the activity of this class of HIV-1 RNase H, we carried out pharmacophore model generation.

**Figure 6:**
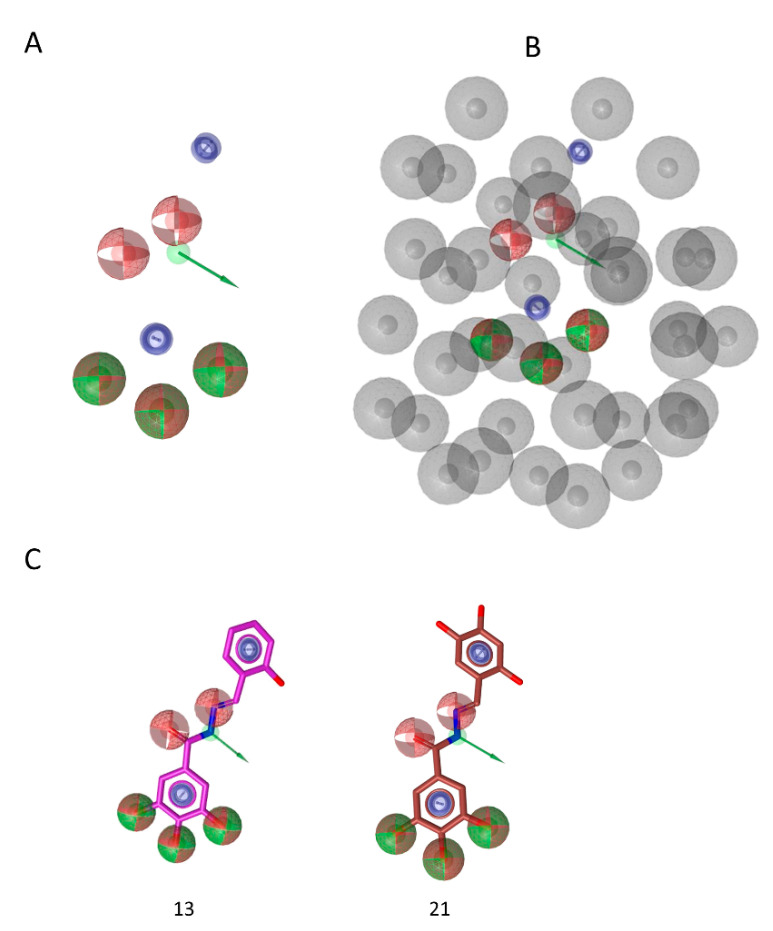
(**A**) Share ligand-based pharmacophore model; (**B**) Share-based pharmacophore model with excluded volumes; (**C**) pharmacophore matching of compound **13** and **21.** Red sphere, H bond acceptor; green sphere and arrow, H bond donor; blue circle, aromatic feature.

The share ligand-based pharmacophore model reported several common features required for inhibitory activity (Figure 6): the hydroxylic groups are key features and their presence in position 3,4,5 of phenyl ring (e.g., compounds **13** and **21**, Figure 6C) is important. In fact, (i) the lack of this portion (e.g., compound **1**), (ii) the hydroxylic group in other positions (compound **5**) or (iii) the substitution with methoxy groups (e.g., compound **19**) led to the loss of activity. Moreover, the presence in the linker of a moiety able to accept and donate hydrogen bonds is relevant, as it is a second aromatic group. These additional features indicate the additional interactions established by the “tail” of the compound while accommodated between alpha helix E and beta sheet 1, that stabilize the complex with the enzyme and are important to the binding, besides the driving force represented by metal binding. While this second aromatic group is a key factor for activity, there are no indications on its possible substitutions. Worth to note, most of the active compounds have hydroxylic group in position 2 (e.g., compounds **13**, **14**, **15**, **16**, **17**, **18**, **20**, **21**, **22**), but others do not have it and their activity is still good (compounds **11**, **12**, **23**), hence suggesting that this group is not essential for interaction with the enzyme. Furthermore, the excluded volume coat (in gray, Figure 6B), highlights the importance of steric occupancy and ideally defines the pocket. Docking experiments (Figure 3) also show that, while pyrogallol moiety drives the binding in the RNase H domain by chelating the ions, the tail is accommodated between alpha helix E and beta sheet 1. Hence the tail cannot be too bulky.

## 4. Discussion

Among the many molecules identified as HIV-1 RT-associated RNase H inhibitors the most potent inhibitors act by coordinating the ionic cofactors within the RNase H active sites [20,23]. Among them, a number of compounds were deeply investigated for their activity on RTs and viruses carrying mutations conferring resistance to approved inhibitors. Results showed that they retained full potency of inhibition against many drug-resistant viruses [10,57,58]. The development of these inhibitors, however, was hampered by the steep substrate barrier [25] or by loss of potency in cell-based assays [22].

Here, we analyzed the mode of action of an active-site RNase H inhibitor, compound **13**, also analyzing the conserved patterns of HIV-1 RT RNase H domain suitable to be targeted to improve its potency.

Cell-based assays showed that compound **13** inhibits the HIV-1 replication with an EC_50_ value of 10.1 μM, in a five-day method with a full replicating virus in MT-4 cells, comparable with other RNase H inhibitors recently reported [22]. Of note, the inhibition of viral replication well reflects compound **13** efficacy in biochemical assays (HIV-1 RNase H IC_50_ = 6.8 μM).

We determined that reverse transcription is the actual target for compound **13**, consistently with the inactivity against HIV-1 IN previously reported [30] and we showed that its activity cannot be correlated with the poor inhibition of HIV-1 RDDP activity (HIV-1 RDDP IC_50_ = 81 μM), possibly caused by some long-range effects already hypothesized for inhibitors binding within the RNase H domain [58]. Consistently, compound **13** retained full potency of inhibition against the NNRTI drug-resistant HIV-1 strain K103N-Y181C.

Despite a relatively low SI, this information encouraged the study of compound **13** binding mode by docking calculations and its validation by site-directed mutagenesis studies. Compound **13**, contains a 3,4,5-trihydroxybenzohydrazone scaffold that shares remarkable similarities with other *N*-acylhydrazones previously reported as the first allosteric inhibitors of HIV-1 RT-associated RNase H function [59,60]. Our binding mode suggested that compound **13** binds within the RNase H active site with the pyrogallol group is serving as the chelating portion, similarly to what is hypothesized for other pyrogallol-derivatives RNase H inhibitors [61]. Compound **13** displayed an orientation different from the one reported for others active site RNase H inhibitors, whose binding mode involved mostly the highly conserved residue His539 [23,58,62] and from the diketo acid inhibitor **RDS1759**. Similar to **RDS1759**, compound **13** shows interactions with residues Asn474 and Gln475 involved in the RNase H primer grip motif [26,31] but, besides, it also interacts with Gly444, Arg448, Glu449 and Arg557 amino acid residues, some of them previously investigated and shown to be involved in the binding of RNase H active-site inhibitors [26,27,28].To prove compound **13** binding mode and its most critical interactions with RT, site-directed mutagenesis experiments were conducted, generating RTs carrying R448A, Q475A or R557A amino acid substitutions. All the three substitutions impair the catalytic efficiency of HIV-1 RT-associated RNase H activity by affecting both kcat and Km values leading to a reduced enzymatic efficiency [26,31].

The inhibitory activity of compound **13** and **RDS1759** was assessed in conditions of competition with the substrate, proving that the compound potency was not affected by substrate displacement, as suggested elsewhere for other classes of compounds [25,63]. The significant loss of potency of compound **13** against all the generated mutant RTs proved the critical role of those amino acid residues for its binding to the RNase H domain and implies a binding orientation different from **RDS1759**, imidazolidinedione derivatives and beta-thujaplicinol [26,27] that were reported to be fully active against both R448A and R557A HIV-1 RTs.

Moreover, to provide useful information not only for the optimization of these molecules, but, in a broader context, for the design of new inhibitors, we performed the analysis of the conservation of the residues of the HIV-1 RT RNase H domain among the circulating variants, using the HIV-1 SGS Database of the Stanford University HIV database [50,51] that contains sequences of isolated from naïve and drug-experienced samples. The analysis revealed a high degree of conservation of the whole HIV-1 RT RNase H domain, particularly regarding the active site catalytic core. Interestingly, the analysis also revealed that more peripheric residues, such as Arg448 and Arg557, involved in the binding of compound **13**, retain a good degree of conservation among naïve patients and patients treated with different classes of RT inhibitors, also carrying drug-resistant mutations [50,51], hence supporting the development of new RNase H inhibitors that would be potentially endowed with high drug-resistant barrier. This could be one of the main reason to justify the development of compounds against a virus with a wide therapeutic armamentarium already available such as HIV, because it may envision to a combination treatment including a lower number of drugs.

Finally, to summarize the structural features required for this new class of active-site HIV-1 RT RNase H inhibitors a pharmacophore model was obtained. The model highlights the chelating moiety already known to be the main feature for other classes of RNase H active site inhibitors such hydroxypyrimidine [20], beta-thujaplicinol derivatives [22,55], *N*-hydroximides [64]. Besides that, the pharmacophore also gave relevant knowledge on the importance and required spatial orientation of aromatic and hydrazone moieties. Of note, the model shows that the coat of the excluded volumes that delimitate the space is also relevant to the design of new molecules.

## 5. Conclusions

We characterized a new promising *N*-acylhydrazone derivative, compound **13**, that selectively inhibits the HIV-1 RT-associated RNase H function during the HIV-1 replication, interacting with highly conserved residues within the RNase H domain of HIV-1 RT and that retains activity against an NNRTI-resistant strain. The binding mode showed different orientations form others previously reported suggesting new possibilities for targeting the highly conserved HIV-1 RT RNase H domain with drugs with potential high-genetic barrier. We also determined a pharmacophore model to understand the important features for the activity and can be used to drive the optimization of the series of molecule analyzed. Furthermore, it can be applied to select new scaffolds in commercial or other available databases. Overall, we have good bases to generate a new series of more potent, HIV-1 RT RNase H selective inhibitors, active against circulating drug-resistant variants, improving their binding and their safety profile.

## Figures and Tables

**Table 1:** Antiviral activity of compounds against wt NL4-3 and NNRTI-resistant HIV-1 strains.

	HIV-1 wt MT EC_50_^1^ (µM)	HIV-1 K103N-Y181C MT4 EC_50_^1^ (µM)	MT4 CC_50_^2^ (µM)	SI ^3^
**13**	10.1 ± 4.7	12.4 ± 0.8	61.5 ± 2.2	6.1
**21**	5.0 ± 1.4	8.7 ± 0.1	31.8 ± 0.4	6.4
**RDS1759**	8.2 ± 0.9	4.6 ± 1.1	39 ± 4.4	4.7
**AZT**	0.007 ± 0.005	0.0031 ± 0.0007	>3.7	>500
**EFV**	0.050 ± 0.012	>1.58	>0.31	>6.25
**NVP**	0.67 ± 0.22	>3.7	>3.7	>5.5
**RAL**	0.0022 ± 0.0001	0.004 ± 0.001	>2.25	>1987

^1^ EC_50_: effective concentration 50 or concentration of compound required to inhibit 50% of HIV-induced cell death, evaluated with the MTT method in MT4 cells; ^2^ CC_50_: cytotoxic concentration 50 or concentration of compound required to induce 50% of death of non-infected cells, evaluated with the MTT method in MT4 cells; ^3^ SI: selectivity index, indicates the ratio between the CC_50_ and the EC_50_; AZT: azidothymidine, EFV: efavirenz, NVP: nevirapine, RAL: raltegravir; Data represent mean and SD of three independent experiments.

## Supplementary Materials

The following are available online at https://www.mdpi.com/1999-4915/12/7/729/s1. Table S1: Percentage of conservation of the amino acid residue in the corresponding position, Table S2: Structures and anti-HIV-1 RT RNase H activities of compounds **1**–**23**.
