# Supplementary material for: Targeting HIV-1 RNase H: N’-(2-Hydroxy-benzylidene)-3,4,5-Trihydroxybenzoylhydrazone as Selective Inhibitor Active against NNRTIs-Resistant Variants
Source: Viruses. 2020 Jul 6;12(7):729. doi: 10.3390/v12070729 (PMC7412550; doi:10.3390/v12070729)
Supplement: Supplementary file 1 [file viruses-12-00729-s001.pdf]

viruses

MDPI

*Supplementary material*

# Targeting HIV-1 RNase H: *N′*-(2-Hydroxy-benzylidene)-3,4,5-Trihydroxybenzoylhydrazone as Selective Inhibitor Active against NNRTIs-Resistant Variants

**Angela Corona [1,*], Ester Ballana [2], Simona Distinto [1], Dominga Rogolino [3], Claudia Del Vecchio [4], Mauro Carcelli [3], Roger Badia [2], Eva Riveira-Muñoz [2], Francesca Esposito [1], Cristina Parolin [4], José A. Esté [2,5], Nicole Grandi[1] and Enzo Tramontano [1,6]**

[1] Department of Life and Environmental Sciences University of Cagliari, Cittadella Universitaria di Monserrato, 09042 Monserrato, Italy
[2] AIDS Research Institute – IrsiCaixa, 08916 Badalona, Spain
[3] Department of Chemistry, Life Sciences and Environmental Sustainability, University of Parma, 43124 Parma, Italy
[4] Department of Molecular Medicine, University of Padova, 35122 Padova, Italy
[5] CienciaTraducida, 08391 Barcelona, Spain
[6] Istituto di Ricerca Genetica e Biomedica, Consiglio Nazionale delle Ricerche (CNR), 09042 Monserrato, Cagliari, Italy
* Correspondence: angela.corona@unica.it; Tel.: +39-070-6754530

**Table S1.** Percentage of conservation of the amino acid residue in the corresponding position.

| HIV-1 RT position | Amino acid | % of conservation |
|---|---|---|
| 427 | Y | 100.00 |
| 428 | Q | 100.00 |
| 429 | L | 99.90 |
| 430 | E | 99.80 |
| 431 | K | 96.20 |
| 432 | E | 77.00 |
| 433 | P | 100.00 |
| 434 | I | 98.20 |
| 435 | V | 35.60 |
| 436 | G | 95.60 |
| 437 | A | 84.00 |
| 438 | E | 99.90 |
| 439 | T | 99.90 |
| 440 | F | 99.30 |
| 441 | Y | 99.90 |
| 442 | V | 100.00 |
| 443 | D | 99.80 |
| 444 | G | 99.90 |
| 445 | A | 100.00 |
| 446 | A | 93.90 |
| 447 | S | 9.00 |
| 448 | R | 96.70 |
| 449 | E | 95.40 |
| 450 | T | 97.70 |
| 451 | K | 98.20 |
| 452 | L | 59.60 |
| 453 | G | 99.80 |

| | | |
|---|---|---|
| 454 | K | 95.90 |
| 455 | A | 100.00 |
| 456 | G | 99.90 |
| 457 | Y | 100.00 |
| 458 | V | 98.20 |
| 459 | T | 99.90 |
| 460 | N | 11.20 |
| 461 | R | 79.50 |
| 462 | G | 99.90 |
| 463 | R | 97.20 |
| 464 | Q | 99.60 |
| 465 | K | 98.40 |
| 466 | V | 67.10 |
| 467 | V | 75.70 |
| 468 | T | 12.10 |
| 469 | L | 91.00 |
| 470 | T | 87.20 |
| 471 | D | 47.50 |
| 472 | T | 99.90 |
| 473 | T | 100.00 |
| 474 | N | 99.90 |
| 475 | Q | 100.00 |
| 476 | K | 95.70 |
| 477 | T | 76.10 |
| 478 | E | 99.80 |
| 479 | L | 100.00 |
| 480 | Q | 82.90 |
| 481 | A | 100.00 |
| 482 | I | 99.50 |
| 483 | H | 36.30 |
| 484 | L | 98.00 |
| 485 | A | 100.00 |
| 486 | L | 100.00 |
| 487 | Q | 99.80 |
| 488 | D | 99.80 |
| 489 | S | 99.90 |
| 490 | G | 100.00 |
| 491 | L | 37.80 |
| 492 | E | 98.90 |
| 493 | V | 99.60 |
| 494 | N | 100.00 |
| 495 | I | 98.40 |
| 496 | V | 99.40 |
| 497 | T | 100.00 |
| 498 | D | 100.00 |
| 499 | S | 99.90 |
| 500 | Q | 99.90 |
| 501 | Y | 99.90 |
| 502 | A | 97.40 |
| 503 | L | 99.70 |
| 504 | G | 99.90 |
| 505 | I | 99.70 |

| | | |
|---|---|---|
| 506 | I | 99.90 |
| 507 | Q | 98.80 |
| 508 | A | 97.60 |
| 509 | Q | 95.60 |
| 510 | P | 100.00 |
| 511 | D | 99.90 |
| 512 | Q | 2.90 |
| 513 | S | 99.90 |
| 514 | E | 96.40 |
| 515 | S | 99.10 |
| 516 | E | 96.70 |
| 517 | L | 88.80 |
| 518 | V | 99.90 |
| 519 | N | 69.10 |
| 520 | Q | 94.80 |
| 521 | I | 99.70 |
| 522 | I | 99.90 |
| 523 | E | 99.80 |
| 524 | Q | 83.20 |
| 525 | L | 99.90 |
| 526 | I | 100.00 |
| 527 | K | 67.70 |
| 528 | K | 99.90 |
| 529 | E | 94.30 |
| 530 | K | 60.80 |
| 531 | V | 69.60 |
| 532 | Y | 99.90 |
| 533 | L | 99.80 |
| 534 | A | 37.10 |
| 535 | W | 99.90 |
| 536 | V | 99.90 |
| 537 | P | 99.90 |
| 538 | A | 99.90 |
| 539 | H | 100.00 |
| 540 | K | 99.90 |
| 541 | G | 99.70 |
| 542 | I | 99.50 |
| 543 | G | 96.40 |
| 544 | G | 99.80 |
| 545 | N | 99.90 |
| 546 | E | 99.30 |
| 547 | Q | 96.60 |
| 548 | V | 94.90 |
| 549 | D | 99.70 |
| 550 | K | 99.70 |
| 551 | L | 99.90 |
| 552 | V | 99.90 |
| 553 | S | 99.90 |
| 554 | A | 100.00 |
| 555 | G | 99.70 |
| 556 | I | 99.10 |
| 557 | R | 99.80 |

| | | |
|---|---|---|
| 558 | K | 87.00 |
| 559 | I | 7.40 |
| 560 | L | 100.00 |

**Table S2.** Structures and anti-HIV-1 RT RNase H activities of compounds **1–23**.

| Compound | Structure | HIV-1 RNase H $IC_{50}$ (μM) |
|---|---|---|
| 1 | 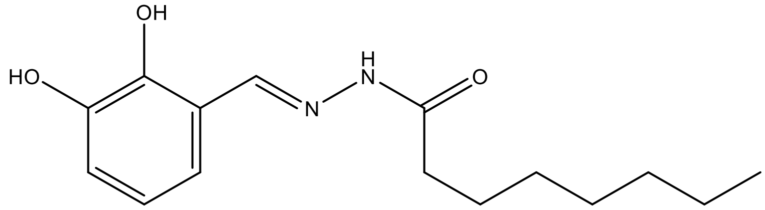 | 61 ± 11 |
| 2 | 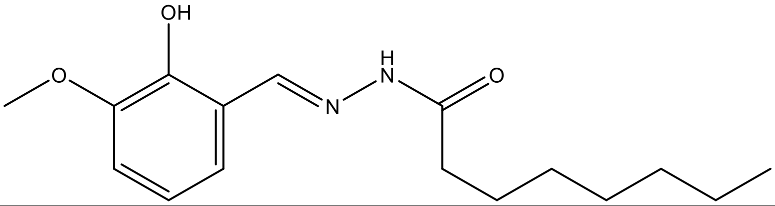 | 62 ± 2 |
| 3 | 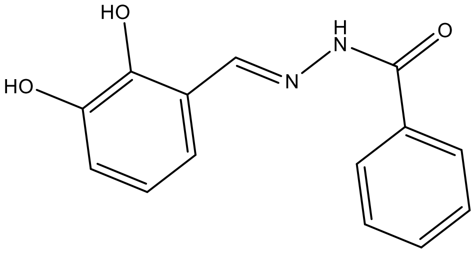 | >100 |
| 4 | 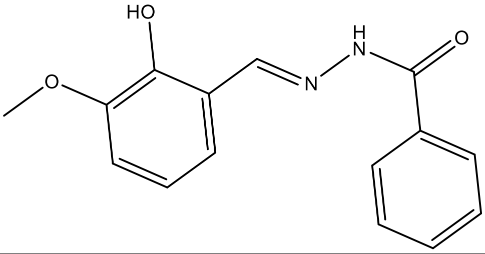 | >100 |
| 5 | 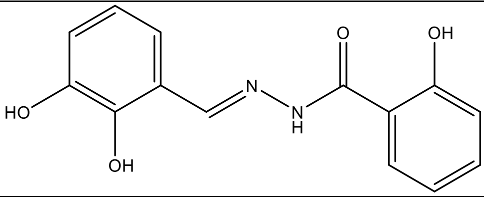 | >100 |
| 6 | 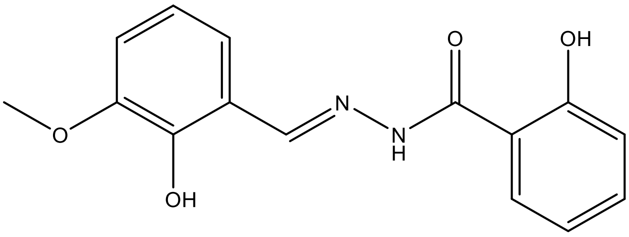 | 37 ± 5 |

| | | |
|---|---|---|
| 7 | 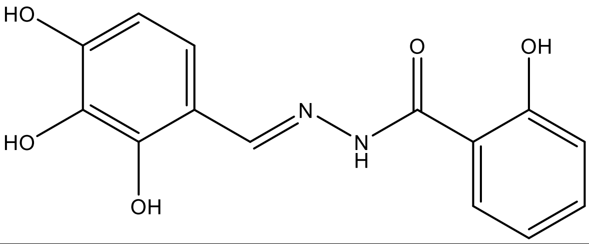 | 25.26 ± 7.56 |
| 8 | 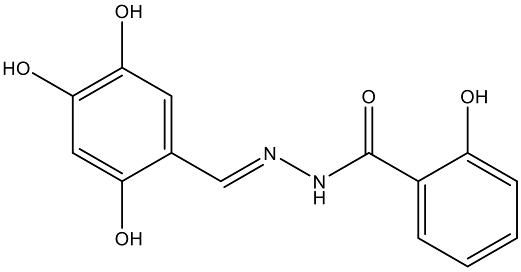 | 8.77 ± 3.13 |
| 9 | 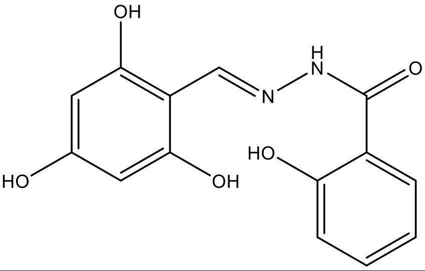 | 9.58 ± 3.43 |
| 10 | 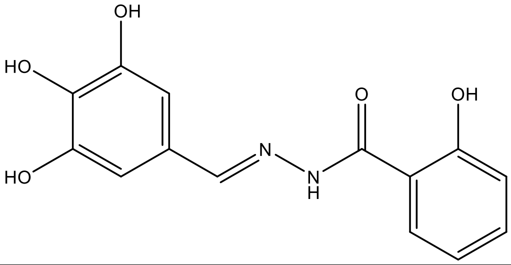 | 4.12 ± 0.22 |
| 11 | 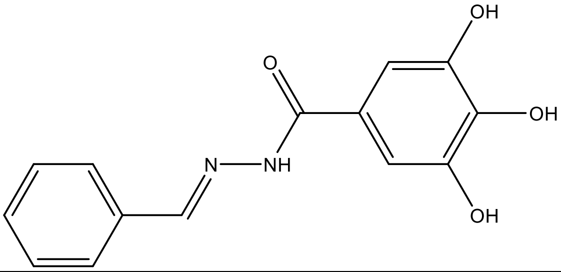 | 2.60 ± 0.59 |
| 12 | 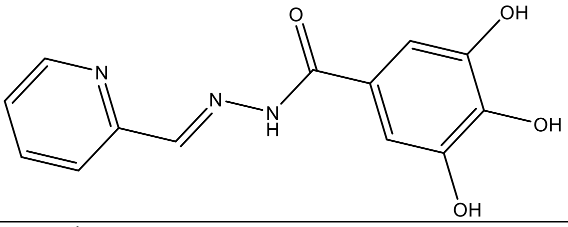 | 3.22 ± 0.54 |
| 13 | 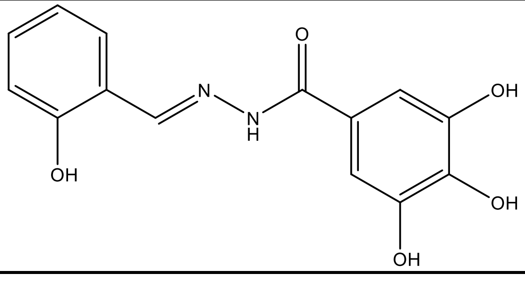 | 2.32 ± 0.75 |

| | | |
|---|---|---|
| 14 | 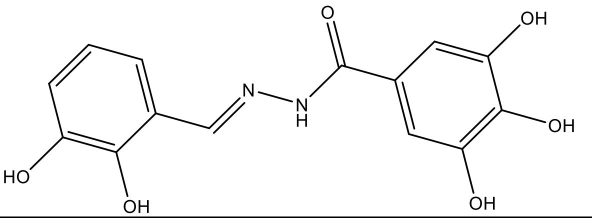 | 3.11 ± 0.47 |
| 15 | 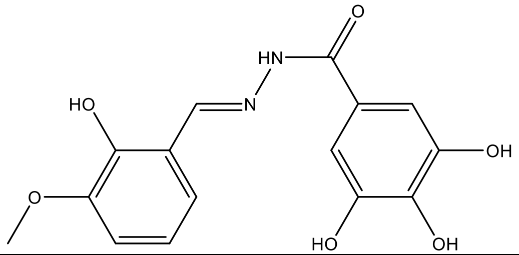 | 0.92 ± 0.02 |
| 16 | 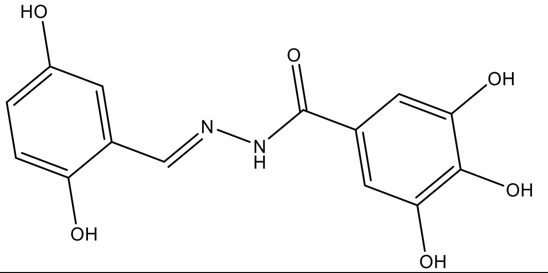 | 2.15 ± 0.11 |
| 17 | 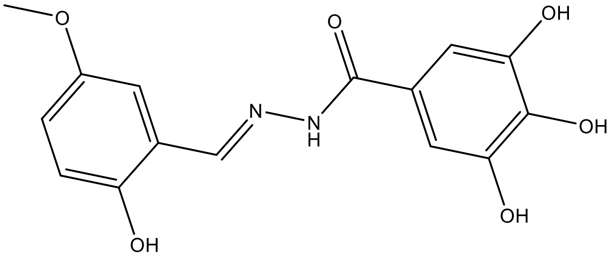 | 2.03 ± 0.59 |
| 18 | 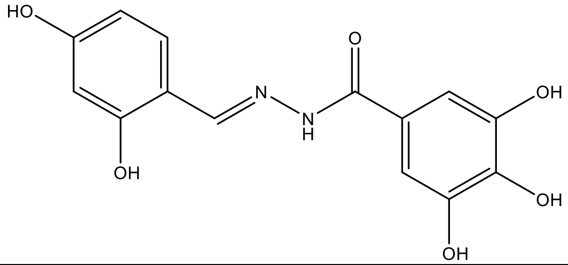 | 1.73 ± 0.07 |
| 19 | 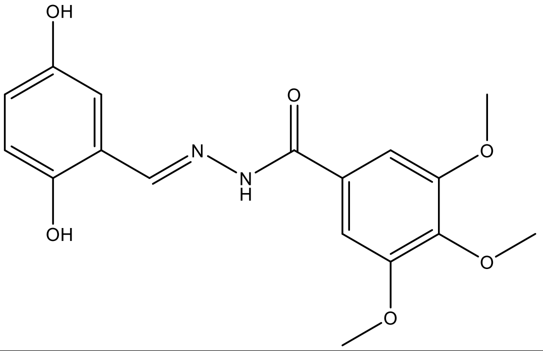 | >100 |

| | | |
|---|---|---|
| 20 | 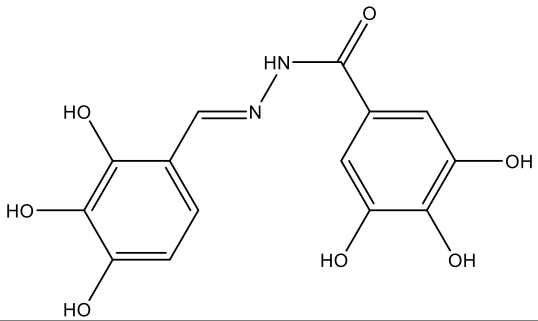 | 0.175 ± 0.002 |
| 21 | 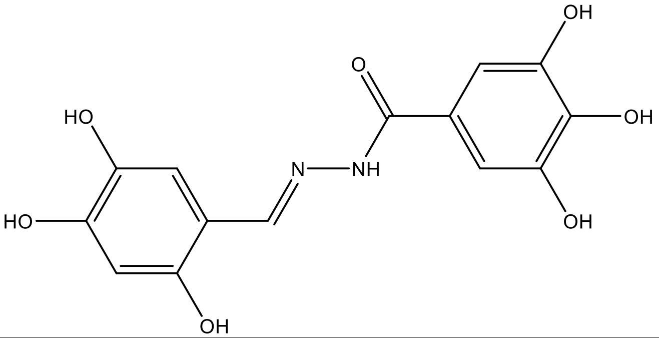 | 0.62 ± 0.10 |
| 22 | 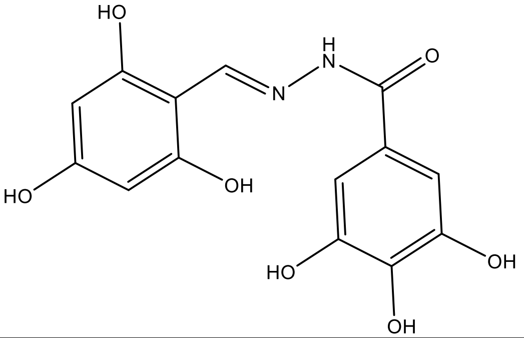 | 9.71 ± 1.57 |
| 23 | 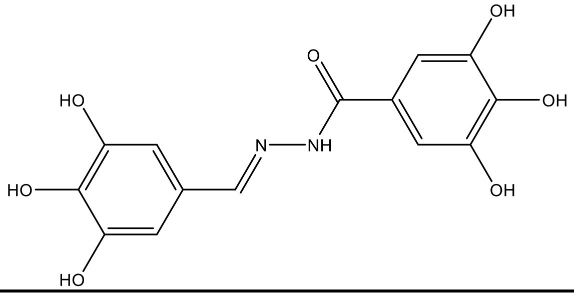 | 0.18 ± 0.03 |

© 2020 by the authors. Submitted for possible open access publication under the terms and conditions of the Creative Commons Attribution (CC BY) license (http://creativecommons.org/licenses/by/4.0/).
